# Stromal IFN-γR-Signaling Modulates Goblet Cell Function During *Salmonella* Typhimurium Infection

**DOI:** 10.1371/journal.pone.0022459

**Published:** 2011-07-28

**Authors:** Pascal Songhet, Manja Barthel, Bärbel Stecher, Andreas J. Müller, Marcus Kremer, Gunnar C. Hansson, Wolf-Dietrich Hardt

**Affiliations:** 1 Institute of Microbiology (D-BIOL), Eidgenössische Technische Hochschule Zürich, Zürich, Switzerland; 2 Institut für Allgemeine Pathologie und Pathologische Anatomie, Technische Universität München, Munich, Germany; 3 Department of Medical Biochemistry, University of Gothenburg, Gothenburg, Sweden; Charité-University Medicine Berlin, Germany

## Abstract

Enteropathogenic bacteria are a frequent cause of diarrhea worldwide. The mucosal defenses against infection are not completely understood. We have used the streptomycin mouse model for *Salmonella* Typhimurium diarrhea to analyze the role of interferon gamma receptor (IFN-γR)-signaling in mucosal defense. IFN-γ is known to contribute to acute *S*. Typhimurium diarrhea. We have compared the acute mucosal inflammation in IFN-γR^-/-^ mice and wild type animals. IFN-γR^-/-^ mice harbored increased pathogen loads in the mucosal epithelium and the lamina propria. Surprisingly, the epithelium of the IFN-γR^-/-^ mice did not show the dramatic “loss” of mucus-filled goblet cell vacuoles, a hallmark of the wild type mucosal infection. Using bone marrow chimeric mice we established that IFN-γR-signaling in stromal cells (e.g. goblet cells, enterocytes) controlled mucus excretion/vacuole loss by goblet cells. In contrast, IFN-γR-signaling in bone marrow-derived cells (e.g. macrophages, DCs, PMNs) was required for restricting pathogen growth in the gut tissue. Thus IFN-γR-signaling influences different mucosal responses to infection, including not only pathogen restriction in the lamina propria, but, as shown here, also goblet cell function.

## Introduction

*Salmonella enterica* subspecies 1 serovar Typhimurium (*S*. Typhimurium) is a frequent foodborne pathogen worldwide [1]. Via virulence factors, encoded on *Salmonella* Pathogenicity Island 1, *S.* Typhimurium induces mucosal inflammation and diarrhea by triggering specific cytokine-networks including pronounced induction of IFN-γ, TNF-α, CXCL2, IL-1b, IL-17, IL-22 and numerous other cytokines [2]–[4]. Although the pathogen exploits these host responses [5], [6], cytokine-signaling is essential for the host for controlling and resolving the infection. Humans with primary immunodeficiency (PID) are highly susceptible to bacterial infections [7]. One key cytokine controlling bacterial infection, i.e. salmonellosis, is interferon gamma (IFN-γ). It controls the systemic spread of the pathogen and contributes to mucosal inflammation [3], [8]–[11].

IFN-γ is produced in the gut and spleen soon after infection [10]–[16], mainly by NK, NKT, CD4^+^ and CD8^+^ T cells [17], and boosts microbial killing by macrophages [18]–[21]. At systemic sites IFN-γ helps to limit bacterial expansion at all stages of the *Salmonella* infection [16], [22]–[25]. Neutralizing anti-IFN-γ antibodies exacerbate the disease [16], [24] and cause relapses in chronically infected mice [23]. Accordingly, IFN-γR^-/-^ mice are hyper-susceptible to systemic S. Typhimurium infection [25], [26].

Mucosal *S.* Typhimurium infections are also affected by IFN-γ. In the streptomycin mouse model, IFN-γ deficiency was shown to result in reduced gut tissue inflammation, reduced T-cell infiltration and reduced induction of MPO, TNFα, CXCL9, CXCL10 MHC-II and VCAM-1 [9], [10], [27]. This is supported by data from PARP1^-/-^ mice which show a delayed IFN-γ response and delayed cecal inflammation [11]. However, besides these global effects, it has remained unclear which cell types of the infected mucosa might be affected by the IFN-γ response. In this paper, we have focused in particular on goblet cells.

Goblet cells are mucin-producing specialized epithelial cells in the small and the large intestine. Mucins, a class of high molecular weight glycoproteins [28], are stored within vacuoles. During homeostasis, continuous secretion occurs at the apical site where the mucins form the mucus gel [29], [30] which (partially) protects against *Yersinia enterocolitica* [31], *Shigella flexneri* [32] and *Citrobacter rodentium* [33].

The major secreted mucin is Muc2 [29], [34]. This glycoprotein is constantly secreted into the lumen where it helps to form a protective gel-like structure. In the colon, this 150 µm thick structure consists of a loose outer layer providing nutrients to commensals, thus fortifying colonization resistance [35], and a tightly packed inner layer, serving as an anchor for sIgA and prohibiting bacterial access to the epithelial surface [33], [36]–[39]. Upon activation by environmental stress or infection, mucin secretion is accelerated, thus reducing the number of mucin-filled vacuoles present in the affected epithelium [40]–[42]. In helminth infections, mucin secretion by the intestinal epithelium is controlled by the Th2 cytokines IL-4 and IL-13 [43]–[45]. However, the regulation of mucin secretion in response to other enteropathogens is still not fully understood [46].

Here, we analyzed mucosal responses coordinated by IFN-γR-signaling during acute bacterial mucosal infection. For this purpose, we used the streptomycin mouse model for *S*. Typhimurium diarrhea. This revealed that IFN-γR-signaling influences at least two different responses of the infected mucosa, namely restriction of pathogen loads in the mucosal tissue, as well as the generation of mucus-filled vacuoles by goblet cells and mucus release into the gut lumen.

## Materials and Methods

### Ethics Statement

All animals were handled in strict accordance with good animal practice as defined by the relevant national and/or local animal welfare bodies, and all animal work was approved by the appropriate committee (Kantonales Veterinäramt Zürich, Zürich Switzerland, license number 201/2007).

### Bacterial strains

SL1344 was utilized as wild type *Salmonella* Typhimurium [47] . For infection, bacteria were cultured in 0.3 M NaCl for 12 h at 37°C and sub-cultivated for 4 hrs as described previously [48]. For detection of bacteria within mucosal tissue, bacteria harbored the reporter plasmid pM973 (*ssaH* promoter fused to *gfp*; [49]).

### Mice

IFN-γR^-/-^ (B6.129S7-Ifngr1^tm1Agt^/J; C57BL/6 background; [50]), C57BL/6ptprc^a^ (congenic marker Ly5.1^+^; [51]) and C57BL/6ptprc^b^ (congenic marker Ly5.2^+^; originally from Charles River) as well as IFN-γR^+/−^ mice (F1 crossing of IFN-γR^-/-^ and C57BL/6ptprc^b^ mice) were kept and bred under specified pathogen free (SPF) conditions at the RCHCI, ETH Zürich. IFN-γR^-/-^ (129-Ifngr^tm1Agt^/J; 129Sv/Ev background; [50]) and 129Sv/Ev mice were bred and obtained from the research contract company (Füllinsdorf, Switzerland). For experiments mice were age (8–12 weeks old) and sex matched and treated as described previously [52], [53]. Briefly, mice were pre-treated with streptomycin (1 dose, 25 mg/animal, by gavage). 24 h later mice were sacrificed (0 dpi) or infected with 5×10^7^ colony forming unit (cfu) by gavage. Infections were performed for 24 h (1 dpi) and 48 h (2 dpi) as well as 96 h (4 dpi). Bacterial loads of gut lumen content, mesenteric lymph nodes (MLN), liver and spleen were determined by plating [53].

### Generation of Bone marrow chimeras

The generation of bone marrow chimeras has been described before [2], [53] and has been approved by the Swiss authorities (license 201/2007 Kantonales Veterinäramt Zürich). In brief, donor mice were euthanatized and bone marrow from femur, tibia, brachium and pelvis were extracted. Recipient mice were γ-irradiated (950 rad) and reconstituted with 3×10^7^ to 6×10^7^ bone marrow cells intravenously. Animals were checked regularly and received drinking water with 0,48% Borgal© (24% solution; Intervet) for 3 weeks. After 8 weeks, reconstitution efficiency was controlled by FACS (Ly5.1/CD45.1, Ly5.2/CD45.2) on spleen and/or blood. In all bone marrow chimeras, the reconstitution efficiency surmounted 90%.

### Histopathological evaluation

Tissues were embedded in OCT (Sakura, Torrance, CA) and snap-frozen in liquid nitrogen. 5 µm cryosections were stained with haematoxylin and eosin. Evaluation was done by a pathologist in a sample-identity-blinded manner, and considered edema, polymorphonuclear (PMN) cell infiltration, loss of goblet cells and epithelium disruption, yielding a score of inflammation between 0–13 points as described recently [2], [52].

### Mucosal tissue colonization

*S*. Typhimurium harbored a reporter plasmid consisting of the *ssaH* promoter fused to *gfp* (pM973; [49]). For evaluating of tissue invaded *S*. Typhimurium, cecum tissue was PFA fixed and stored as descripted before [53]. 20 µm cryosections were stained with Armenian hamster α-ICAM-I/CD54 (clone 3E2) (1∶100; Becton Dickson), DAPI (1∶1000, Sigma-Aldrich), Cy3-conjugated goat α–Armenian hamster IgG (1∶100, Jackson ImmunoResearch Laboratories) and AlexaFluor647 conjugated phalloidin (1∶100, Molecular Probes) [42], [53]. To evaluate the average number of invaded bacteria in the epithelium and lamina propria, 3 tissue sections per mouse were counted.

### Immunofluorescence staining

For Ki67- and CD54-staining, 7 µm cryosections were made from OCT-embedded snap-frozen tissue. Triton pre-treatment and staining procedures have been described previously [2], [54]: polyclonal rabbit α-Ki67 (1∶100; Abcam), Armenian hamster α-ICAM-I/CD54 (clone 3E2) (1∶100; Becton Dickson), FITC-conjugated α-rabbit IgG and Cy3-conjugated goat α–Armenian hamster IgG (each 1∶100, Jackson ImmunoResearch Laboratories), TO-PRO 3 iodide (1∶6000, Molecular Probes) and AlexaFluor350 conjugated phalloidin (1∶100, Molecular Probes). The mucus staining with the lectin UEA-1 has been described recently [42]. 7 µm PFA fixed cryosections were stained with rhodamine conjugated UEA-1 (1∶100, Reactolab), TO-PRO 3 Iodide (1∶6000, Molecular Probes) and Alexa488 conjugated phalloidin (1∶100, Molecular Probes). The staining with the lectin WGA was done similarly to UEA-1: AlexaFluor647 conjugated WGA (1∶100, Molecular Probes), SytoxGreen (1∶10000, Molecular Probes) and TRITC conjugated phalloidin (1∶100, Molecular Probes) were used. For the quantification of WGA^+^ cells, we evaluated three stained 7 µm tissue sections from 5 mice per group (three 20x images per section). Bars represent the mean number of WGA^+^ cells (+/− standard error of mean) in each experimented group. Images were taken with a Zeiss Axiovert 200 m inverted microscope equipped with an Ultraview confocal head (PerkinElmer) and a Plan Neofluoar x20 objective with an aperture setting of 0.55 (Zeiss).

### Muc2^+^ cells

7 µm cryosections of snap-frozen tissue were stained with a polyclonal rabbit antiserum PH497 α-GpA Muc2 [55] that stains the non-glycosylated Muc2-precursors of the goblet cells. As a secondary antibody we employed FITC-conjugated α-rabbit IgG (1∶100, Jackson ImmunoResearch Laboratories) and DAPI (1∶1000, Sigma-Aldrich). 3 mice were analyzed per group. Each bar represents the mean number of Muc2^+^ cells (+/− standard error of mean) of the 20x optical field of three different sections per mouse.

### RT PCR analysis

The cecum tissue was washed in PBS (4°C), submerged in 600 µl RNAlater-buffer (RNeasy Mini Kit, Qiagen). RNA was processed using the RNeasy mini kit (Qiagen), the RNase-Free DNase kit (Qiagen), M-MLV reverse Transcriptase RNase H Minus (Promega) and RNasin Plus (Promega). qPCR analysis of *Gapdh* (GGC TGC CCA GAA CAT CAT CCC TGC AT; ACG TCA GAT CCA CGA CGG ACA CAT TGG), *cxcl2* (AGG CTC CTC CTT TCC AGG TC; CCC CCT GGT TCA GAA AAT CA) and *muc2* (CAA AGG CCT CAC CAC CAA GC; GCG TGG CAC TGG GAG AAT AG) was performed via the FastStart Universal SYBR Green Master (Roche, Switzerland). Relative *cxcl2* and *muc2* mRNA levels (2^-ΔΔCt^) were normalized using the values for *Gapdh*. Cycling parameters were 94°C (15 s), 60°C (30 s), 72°C (30 s) in a RotorGene 3000 cycler (Corbett Research, Cambridgeshire, UK).

### Statistical Analysis

Statistical Analysis has been performed using the exact Mann-Whitney *U*-test and the software GraphPad Prism 5. For the Muc2^+^ cell evaluation, the unpaired t-test (two-tailed) has been used. Values of p<0.05 were considered as significantly different between two groups.

## Results

### Increased number of mucus-filled goblet cells in the infected mucosa of IFN-γR^-/-^ mice

Earlier work had established that acute *S*. Typhimurium infection leads to a dramatic depletion of mucus-filled goblet cells from the cecal mucosa [52], [53]. Pilot experiments indicated that this phenotype was much less pronounced in IFN-γR deficient mice. This was observed in different genetic mouse backgrounds and at different times after infection (Figure S1).

**Figure 1:**
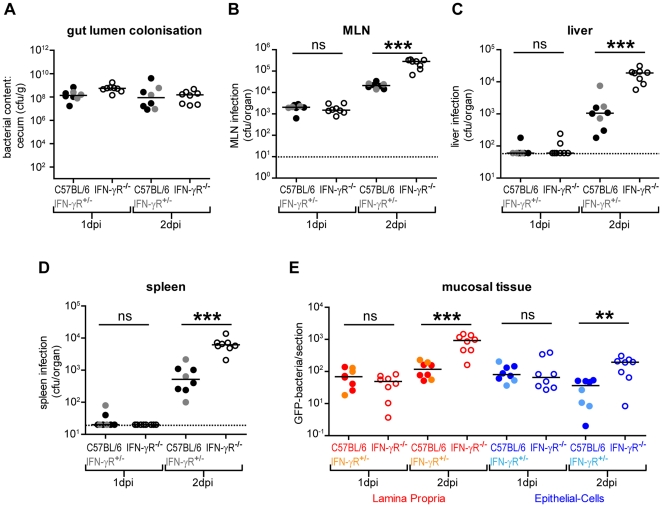
Mucosal *S*. Typhimurium infection in IFN-γR^-/-^ and isogenic control mice. IFN-γR^-/-^ mice (C57BL/6 background; open symbols), C57BL/6 controls (closed black symbols) and IFN-γR^+/−^ control mice (closed grey symbols) were pre-treated with streptomycin and infected for 1 day (1 dpi) or 2 days (2 dpi) with *S*. Typhimurium (SL1344 pM973; Material and Methods). We analyzed colonization levels in the gut lumen (A), the MLNs (B), the livers (C), the spleens (D) and the mucosal tissues (E; red  =  lamina propria; blue  =  epithelial cells). Data point color code: closed symbols (red, dark blue)  =  C57BL/6 mice; closed symbols (orange, light blue)  =  IFN-γR^+/−^ control littermates; open symbols (red, dark blue)  =  IFN-γR^+/−^ mice. *: p<0.05.

To systematically analyze the role of interferon gamma receptor (IFN-γR) signaling in acute *S*. Typhimurium colitis, we employed the streptomycin mouse model. Streptomycin pre-treated IFN-γR^-/-^ mice ([50] n = 8 mice), C57BL/6 controls (n = 5 mice; black circles) and IFN-γR^+/−^ littermate control mice (n = 3 mice; grey circles) were infected with wild type *S*. Typhimurium SL1344 (pM973; 5×10^7^ cfu by gavage, [49]) as described in Materials and Methods. The pathogen harbored the GFP reporter plasmid pM973 which drives *gfp*-expression soon after *S*. Typhimurium has entered into the gut tissue. This allows enumerating pathogen loads in the gut tissue by fluorescence microscopy [49]. Mice were sacrificed at 1 day or 2 days post infection and analyzed for pathogen loads in the cecum lumen, the mesenteric lymph nodes (MLN), the liver and the spleen (Fig. 1A–D). *S*. Typhimurium efficiently colonized the intestinal lumen in all groups of mice (Fig. 1A). The MLN were colonized by day 1 p.i. and pathogen loads did not differ significantly between the control group consisting of wild type and IFN-γR^+/−^ littermates and the IFN-γR^-/-^ mice (approx. 10^3^ cfu/ MLN; p≥0.05; Fig. 1B). However, during the next 24 h, MLN pathogen loads increased more slowly in the control mice than in the knockout animals (approx. 10^5^ vs. 10^4^ cfu/ MLN; p<0.05). Similarly, the pathogen loads in the liver and the spleen of the IFN-γR^-/-^ mice were significantly increased by day 2 p.i. (p<0.05; Fig. 1C,D; open symbols). This was in line with earlier data and indicated that IFN-γR-signaling contributes to controlling infection at systemic sites [16], [22]–[25].

Next, we analyzed the pathogen colonization of the cecal mucosa. At day 1 p.i., we detected equivalent pathogen loads in the cecal lamina propria of wild type (red closed symbols), the IFN-γR^+/−^ (orange closed symbols) and the IFN-γR^-/-^ mice (open red symbols; p≥0.05; approx. 20–100 bacteria per tissue section; Fig. 1E). However, by day 2 p.i., pathogen loads had increased significantly in the lamina propria of the IFN-γR^-/-^ mice, but not in the control animals (p<0.05; Fig. 1E). Similarly, in the epithelial cells the IFN-γR-deficiency had no effect at day 1 p.i., but pathogen loads increased significantly in the IFN-γR^-/-^ mice by day 2 p.i. (p<0.05; Fig. 1E, blue symbols). This indicated that IFN-γR-signaling helps controlling mucosal tissue infection at early time points post infection.

**Figure 2:**
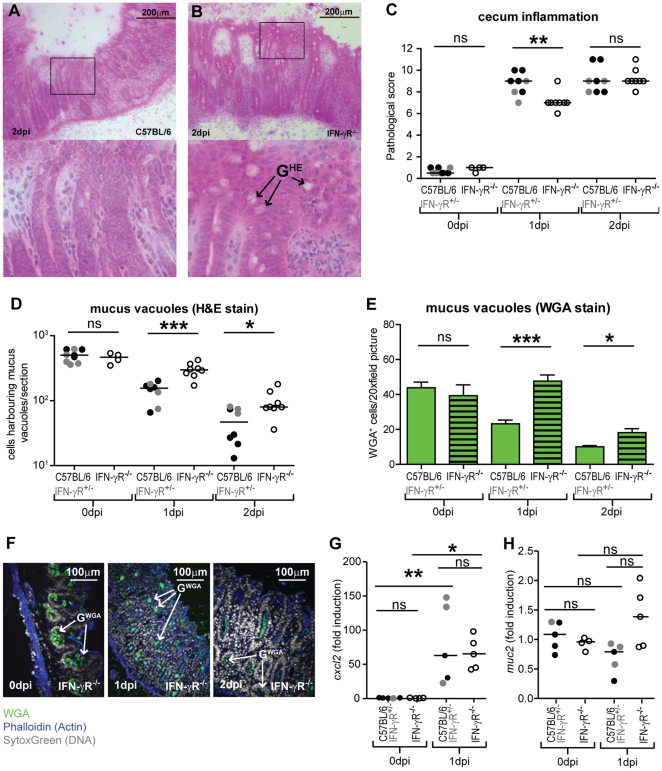
Mucosal pathology of *S*. Typhimurium infected IFN-γR^-/-^ mice. Infected animals from Fig. 1 (IFN-γR^-/-^ mice, open symbols; C57BL/6 control, closed black symbols; and IFN-γR^+/−^ control mice, closed grey symbols) as well as streptomycin pre-treated non-infected animals (0 dpi) were analyzed (A–F). (A,B) HE-stained tissue sections from wt C57BL/6 controls and IFN-γR^-/-^ mice at day 2 p.i.. (C) Inflammation of the cecal mucosa. (D) The number of goblet cells harboring mucus-filled vacuoles was analyzed using HE-stained tissue sections. (E) WGA-AlexaFluor647 staining for mucin (incl. mucus filled vacuoles, secreted mucins). (F) Quantitative analysis of the number of goblet cells which harbor mucus-filled vacuoles as determined by WGA-AlexaFluor647 fluorescence microscopy (see E). G^HE^: mucus-filled goblet cell vacuoles detected in HE-stained tissue sections. G^WGA^: mucin-filled goblet cell vacuoles detected in tissue sections stained with the lectin WGA-AlexaFluor647. (G, H). Quantitative real time PCR analysis of *cxcl2* and *muc2* expression. RNA samples from IFN-γR^-/-^ mice (open symbols), C57BL/6 control, (closed black symbols) and IFN-γR^+/−^ control mice (closed grey symbols) were selected from the experiment shown above. mRNA expression levels were normalized to *Gapdh* (Materials and Methods). *: p<0.05; ns: not statistically significant. Black line: median. Errors bars (F): standard error of mean; bar  = 100 µm.

Then, we analyzed the role of IFN-γR-signaling with respect to gut inflammation and the goblet cell phenotype, as observed in the experiments above (Fig. 2A–H). In the absence of infection, IFN-γR^-/-^ and wt control mice displayed no signs of mucosal pathology, equivalent numbers of mucus filled goblet cells and equivalent mRNA levels for *muc2*, the gene encoding the key secreted goblet cell mucin (p≥0.05; Fig. 2C,D,F,H; 0 dpi). Upon infection, all mice developed pronounced mucosal inflammation (Fig. 2A–C). At day 1 p.i., cecal pathology (but not *cxcl2*-expression; Fig. 2G) of IFN-γR^-/-^ mice was slightly reduced compared to wild type and IFN-γR^+/−^ littermates. This is in line with previous reports [9], [11]. However, by day 2 p.i. knockout and wild type mice reached equivalent levels of mucosal inflammation. In line with a large body of previous work (e.g. [42], [52]), the wt mice showed severely reduced numbers of mucus-filled goblet cells after infection. In contrast, the cecal mucosa of infected IFN-γR^-/-^ mice retained much higher numbers of mucus-filled vacuoles (Fig. 2A,B; see marked G^HE^ vacuoles in Fig. 2B, right panel). This confirmed date presented, above (Figure S1).

WGA-immunofluorescence microscopy confirmed this phenotype (Fig. 2E; see marked G^WGA^ vacuoles in the right panel; [42]). The wheat germ agglutinin (WGA) recognizes glyco-epitopes of the mature, modified mucins stored within the mucus-filled vacuoles of goblet cells. Via WGA-staining we confirmed the presence of higher numbers of mucin-filled goblet cells in the cecal mucosa of IFN-γR^-/-^ compared to C57BL/6 mice and IFN-γR^+/−^ littermates (p<0.05; Fig. 2F). Finally, real time PCR analyses demonstrated that this was not attributable to changes in *muc2* gene expression (p≥0.05; Fig. 2G,H). In conclusion, these data suggested that IFN-γR-signaling affects the kinetics of mucin release by goblet cells in the infected cecal mucosa.

### IFN-γR-signaling in the stromal compartment controls goblet cell function

To determine whether the IFN-γR-mediated control of the goblet cells is conferred by the stromal or the bone marrow derived compartment, we generated bone marrow chimeras (BM chimeras) and challenged the animals in the streptomycin mouse model. We generated three types of BM chimeric animals: a) "B6/IFN-γR" chimeras by reconstituting C57BL/6 mice (Ly5.1 marker) with IFN-γR^-/-^ (Ly5.2 marker) bone marrow; b) "IFN-γR/B6" chimeras by reconstituting IFN-γR^-/-^ mice (Ly5.2 marker) with C57BL/6 (Ly5.1 marker) bone marrow; c) "B6/B6" chimeras by reconstituting C57BL/6 mice (Ly5.2 marker) with C57BL/6 (Ly5.1 marker) bone marrow as described in Materials and Methods.

**Figure 3:**
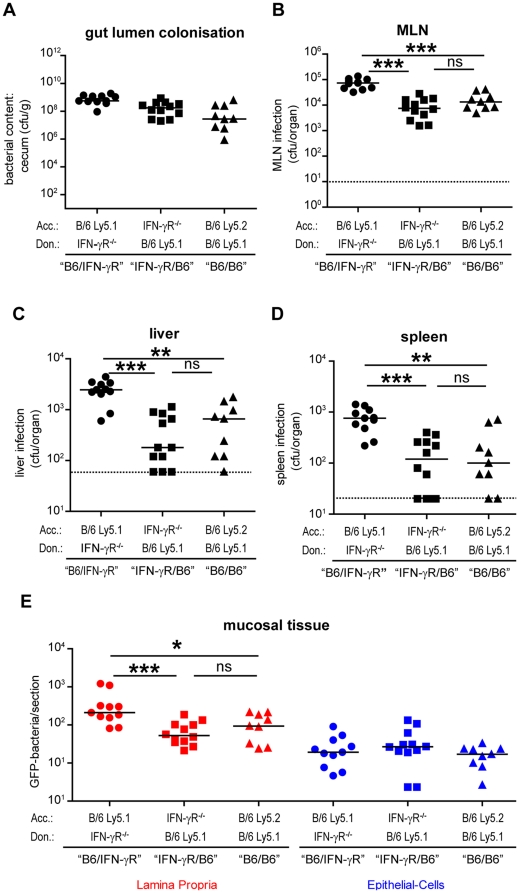
Mucosal *S*. Typhimurium infection in IFN-γR^-/-^ bone marrow chimeras and IFN-γR^+/+^ controls. We generated bone marrow chimeras (BM chimeras) and challenged the animals with *S*. Typhimurium (SL1344; harboring pM973) in the streptomycin mouse model for 2 days (A-E). Types of BM chimeric animals generated: "B6/IFN-γR" [C57BL/6 mice (Ly5.1 marker) reconstituted with IFN-γR^-/-^ (Ly5.2 marker) bone marrow; circles], "IFN-γR/B6" [IFN-γR^-/-^ mice (Ly5.2 marker) reconstituted with C57BL/6 (Ly5.1 marker) bone marrow; squares] and "B6/B6" [C57BL/6 mice (Ly5.2 marker) reconstituted with C57BL/6 (Ly5.1 marker) bone marrow; triangles] . We analyzed colonization levels in the gut lumen (A), the MLNs (B), the livers (C), the spleens (D) and the mucosal tissues (E; red  =  lamina propria; blue  =  epithelial cells). *: p<0.05. Black line: median. n.s.: not significant (p≥0.05). Stippled line: minimal detectable value.

The BM chimeric mice were pre-treated with streptomycin and infected with wild type *S*. Typhimurium SL1344 (pM973; n = 9 to 12 mice per group; 5×10^7^ cfu by gavage). Mice were sacrificed at day 2 p.i. and we analyzed pathogen loads in the cecum lumen, the mesenteric lymph nodes (MLN), the liver and the spleen (Fig. 3A–D). *S*. Typhimurium efficiently colonized the intestinal lumen in all groups of mice (Fig. 3A). The "B6/IFN-γR" chimeras harbored significantly higher pathogen loads in the MLN, the liver and the spleen than the "IFN-γR/B6" chimeras or the "B6/B6" control chimeras (p<0.05; Fig. 3B,C,D). This indicated that in cells of the BM-derived compartment (i.e. macrophages, PMNs, DCs) IFN-γR-signaling is essential for controlling pathogen spread to systemic sites, while IFN-γR-signaling in stromal cells is dispensable.

Next, we analyzed the infection of the cecal mucosa. Equivalent pathogen loads were detected in the epithelial cells from all three groups of mice (p≥0.05; Fig. 3E, blue symbols). In contrast, the lamina propria pathogen loads were significantly higher in the "B6/IFN-γR" chimeras, than in the "IFN-γR/B6" chimeras or the "B6/B6" controls (p<0.05; Fig. 3E, red symbols). Thus, IFN-γR-signaling in cells of the BM-derived compartment (i.e. macrophages, PMNs, DCs) is required normally to control pathogen burdens in the cecal mucosa, while IFN-γR-signaling in stromal cells does not affect pathogen loads.

**Figure 4:**
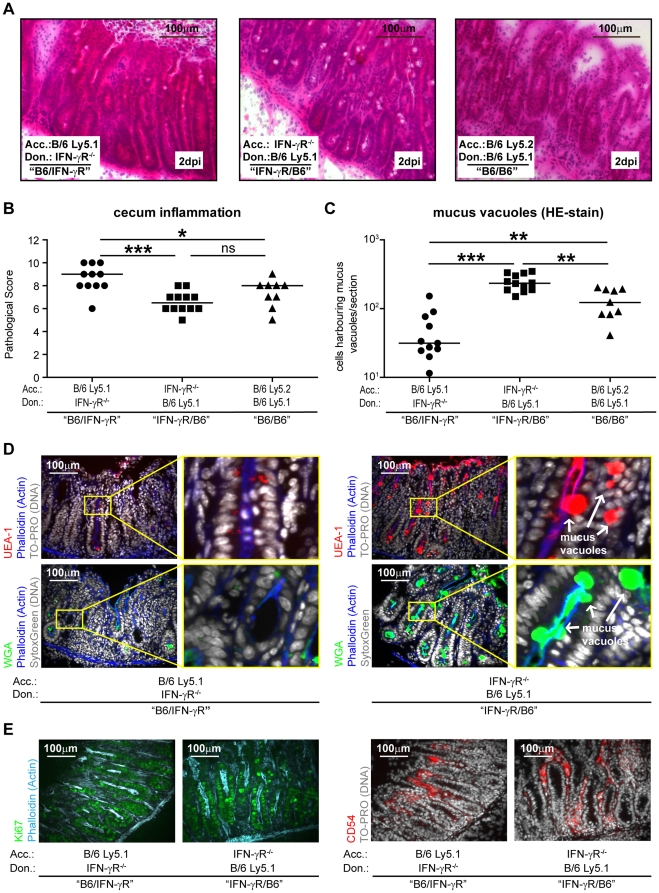
Mucosal pathology and mucus-filled vacuoles in the mucosa of *S*. Typhimurium-infected IFN-γR^-/-^-bone marrow chimeras. We analyzed the cecum mucosa of *S.* Typhimurium-infected BM chimeras shown in Fig. 3. (A), HE-stained gut tissue sections. (B) Mucosal tissue pathology. (C) Quantitative analysis of the number of mucus vacuoles in the cecal mucosa of infected BM chimeras as determined from HE-stained tissue sections. (D) UEA-1-rhodamine or WGA-AlexaFluor647 staining of glycoconjugates /mucins. (E) Staining of the cell-proliferation marker Ki67 and the lamina propria marker CD54 (E). *: p<0.05; Black line: median. n.s.: not significant (p≥0.05). Bar  = 100 µm.

Finally, we evaluated mucosal inflammation. Gut inflammation was slightly more pronounced in the "B6/IFN-γR" chimeras than in the "IFN-γR/B6" chimeras or the "B6/B6" control chimeras (p<0.05; Fig. 4A,B). This was well in line with the increased lamina propria burdens observed in the "B6/IFN-γR" chimeras (Fig. 3E). Then we enumerated the goblet cells harboring mucus-filled vacuoles. The gut tissue of "B6/IFN-γR" chimeras harbored the lowest number of these cells (p<0.05; Fig. 4C). This was in line with the stronger mucosal inflammation and the increased lamina propria burdens observed in these animals. Interestingly, the "IFN-γR/B6" chimeras harbored significantly more mucus filled goblet cells than the "B6/IFN-γR" chimeras or the "B6/B6" controls (p<0.05; Fig. 4C). All other parameters of the mucosal disease were equivalent between the "IFN-γR/B6" chimeras and the "B6/B6" controls (p≥0.05; Figs. 3 and 4). In conclusion, these data indicated that IFN-γR-signaling in stromal cells modulates mucus secretion by goblet cells.

To further substantiate this observation, we evaluated the infected gut tissues by immunofluorescence microscopy. Besides wheat germ agglutinin (WGA), the lectin *Ulex europaeus* agglutinin-1 (UEA-1) can also recognize glyco-epitopes of the mature, modified mucins stored in goblet cells. Secreted mucins on the epithelial surface and within the gut lumen are also stained by these two lectins (Fig. 4D; [42]). Staining with these markers verified that the mucosa of "IFN-γR/B6" chimeras harbored large numbers of goblet cells with mucus-filled vacuoles (Fig. 4D). Control stains for the lamina propria marker CD54 (ICAM-I; stains lamina propria and most cells of the gut associated immune system) or the proliferation marker Ki67 (a measure of tissue regeneration; see for example [2], [54]) yielded equivalent results for the "IFN-γR/B6" and the "B6/IFN-γR" chimeras (Fig. 4E). These data corroborated that goblet cell function is directly modulated by IFN-γR-signaling, e.g. by controlling the genesis of the mucus-filled vesicles or by triggering mucus release. Thus, IFN-γR-signaling is required on hematopoietic cells to control pathogen burden, and on the stromal compartment to facilitate increased mucus secretion.

### The density of goblet cells is not affected in IFN-γR-deficient mice

**Figure 5:**
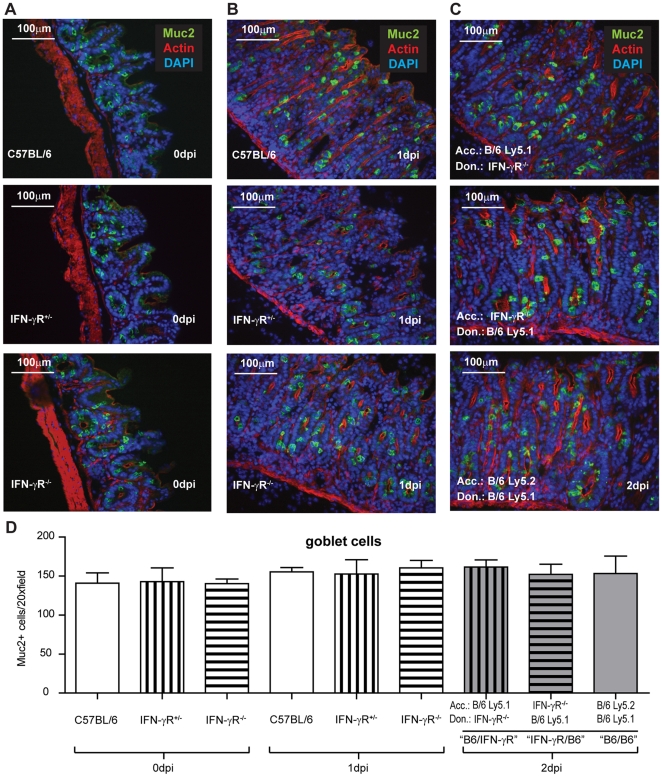
Mucus-filled goblet cell vacuoles in the cecal mucosa of *S*. Typhimurium infected BM chimeras, C57BL/6 controls, IFN-γR^+/−^ mice and IFN-γR^-/-^ animals. Cecum tissues of BM chimeras from Fig. 3 and 4 (2 dpi), as well as cecum tissue from C57BL/6 controls, IFN-γR+/− littermates and IFN-γR-/- mice from Fig. 2 (0 dpi and 1 dpi) were analyzed. (A–C) Immunofluorescence microscopy images of cecal tissue sections from non-infected controls (A) and mice at days 1 p.i. (B) and day 2 p.i. (C). Green, anti-Muc2-antibody staining; The Muc2-specific antibody recognizes an epitope of immature mucin, located within goblet cells, but absent from secreted Muc2. red: actin (phalloidin staining); blue: DNA (DAPI staining). (D) Quantitative analysis of the number of goblet cells which harbor mucus-filled vacuoles as determined by Muc2 fluorescence microscopy. No significant differences were observed when compared to non-infected C57BL/6 mice (p≥0.05). Errors bars (D): standard error of mean. Bar  = 100 µm.

Finally, we have addressed whether goblet cell function is affected at the level of mucus secretion. Alternatively, the genesis or maintenance of goblet cells might be affected in IFN-γR-deficient mice. The latter might be suggested by recent findings on the role of IFN-γ in regulating intestinal epithelial homeostasis and function [56], [57]. In order to address this issue, we employed immunofluorescence microscopy and a Muc2-specific antibody. This antibody recognizes specifically an epitope of immature mucin which is always located within goblet cells, but which is not secreted. Thus, the α-Muc2-antibody allows staining of all goblet cells, synthesizing or storing Muc2, independent of the presence or absence of mucus-filled vacuoles [38], [42]. Using the α-Muc2-antibody, we detected equivalent numbers of goblet cells in the cecal mucosa of all BM chimeras ("B6/IFN-γR", "IFN-γR/B6" and "B6/B6; gray shading; p≥0.05; Fig. 5CD). We also analyzed IFN-γR^-/-^ mice and control wild type C57BL/6 mice as well as IFN-γR^+/−^ littermates prior to infection (0 dpi) and at day 1 p.i.. However, no difference in the number of Muc2^+^ cells could be detected between any of the groups, analyzed (Fig. 5 A,B,D). In addition the number of Muc2^+^ cells was also not affected by the procedures for generating the BM chimeras as judged by comparison with non-irradiated groups (C57BL/6, IFN-γR^+/−^, IFN-γR^-/-^ mice at day 0 p.i. and 1 p.i.; p≥0.05; Fig. 5D). These data confirmed that goblet cell numbers were not affected by IFN-γR-deficiency. Rather, IFN-γR-signaling seems to drive the secretion of goblet cell content.

## Discussion

Interferon gamma is a key cytokine coordinating immune defense against infection. Earlier work had established that IFN-γ contributes to acute mucosal inflammation in the streptomycin model for *Salmonella* diarrhea [9]. This is confirmed by our findings in IFN-γR^-/-^ mice (Fig. 2C; 1 day p.i.) and the delayed gut inflammation at 8h p.i. in PARP1^-/-^ and IFN-γ^-/-^ mice ([11], Hapfelmeier and Hardt, unpublished observations). Here, we have extended these studies by demonstrating that IFN-γR-signaling affects the acute mucosal disease in at least two ways, i.e. by restricting pathogen loads in the lamina propria and by controlling mucus accumulation/secretion by goblet cells. The former requires IFN-γR-signaling in BM-derived cells, presumably macrophages, DCs and/or PMNs, while the latter depends on IFN-γR-signaling in stromal cells. Thus, during the acute *S*. Typhimurium infection, different compartments of the intestinal mucosa are functionally coordinated by IFN-γR-signaling.

How does IFN-γR-signaling affect mucosal colonization by *S*. Typhimurium? Earlier work had established that IFN-γR-signaling is essential for controlling the systemic infection by numerous pathogens, including *S*. Typhimurium ([3], [16], [22]-[25] ; this work). This is attributable to the pronounced up-regulation of bactericidal capacities in IFN-γ exposed macrophages and their key role in pathogen elimination at systemic sites. In the intestinal mucosa, *S*. Typhimurium resides in macrophages, but also in other cell types like PMNs, DCs and epithelial cells/enterocytes [2], [49]. Therefore, it was previously unclear whether IFN-γ would have a similar effect on pathogen control as observed at systemic sites. Our work establishes that this is indeed the case. In IFN-γR^-/-^ mice and BM-chimeric mice lacking IFN-γR in BM-derived cells, *S*. Typhimurium can enter the cecal tissue and colonize the lamina propria, without being restricted. Interestingly, increased lamina propria colonization has been observed previously in Cybb/Nos2 knockout mice [58]. These mice lack NADPH oxidase 2 and inducible NO-synthase, two enzymes required for the production of oxygen and nitrogen species which are key anti-microbial agents produced by macrophages and by PMNs [59]. This suggests that IFN-γR-signaling induces these antimicrobial defenses of mucosal phagocytes. Identifying the key cell types and the remaining elements of the signaling pathways eliciting Cybb/Nos2-responses in the intestinal mucosa will be an interesting topic for future research.

The pathogen loads in the cecal epithelium were also increased in the IFN-γR^-/-^ mice (Fig. 1E, day 2 p.i.; open blue symbols). There are at least three different explanations for this observation: 1) Reduced mucus secretion might lower the mucus mediated bacterial clearance from the epithelial surface and result in increased pathogen invasion rates from the gut lumen; 2) the IFN-γR^-/-^ epithelium might fail to restrict pathogen growth within this cell type; 3) Increased pathogen loads in the IFN-γR^-/-^ lamina propria might lead to increased rates of pathogen invasion from the basolateral side. This might be similar to the basolateral epithelium invasion by *Shigella flexneri*, another enteropathogenic bacterium [60]. Interestingly, IFN-γR-deficiency in the stromal- or the BM-derived compartment alone did not increase epithelial pathogen loads (Fig. 3E, blue circles, blue squares). This suggests that multiple mechanisms involving BM-derived and stromal cells cooperate in order to restrict pathogen loads in the intestinal epithelium.

During infection, IFN-γR^-/-^ goblet cells display strikingly high numbers of mucus-containing vacuoles. These numbers are equivalent to those observed under steady state conditions in the healthy mucosa. Based on this observation, it seems most likely that IFN-γ, which is triggered during the first 6h of the gut infection [11], triggers the release of these vacuolar contents. However, we cannot rule out that IFN-γ may affect other parameters of mucus-vacuole formation, like vacuole size or the rate of vacuole formation. Future work will have to address this issue.

Mucus is an important defense against mucosal pathogens. For example, *muc2* ^-/-^ mice are susceptible to chronic mucosal inflammation elicited by the normal gut flora and to lethal infectious colitis by *Citrobacter rodentium* [33], [39]. However, mucin gene expression, mucus release and cytokine control seem to vary significantly between different infection models. For example, cholera toxin is known to elicit mucus secretion and secretion can be inhibited by IFN-γ, as shown in a HT29 Cl.16E human tissue culture model [61]. This differs from our findings in the murine *Salmonella* diarrhea model, where IFN-γ-signaling is required for mucus release. We do not know whether this difference is attributable to the different nature of the two models (tissue culture cell line vs. in vivo). Alternatively, different secretion stimuli (*Salmonella* infection vs. cholera toxin etc.) might differ in their positive/negative modulation by IFN-γ. Also, we cannot exclude species-specific differences in the control of mucin release from goblet cells between humans and mice.

Shekels at al. [62] used a mouse gut infection model to analyze the effect of nematode infection with *Trichinella spiralis* on mucosal *muc2* and *muc3* mRNA levels as well as mucus vacuole size/numbers in the small intestine. Interestingly, in this nematode model, the mucin mRNA levels and the number of goblet cells harboring mucus-filled vacuoles increased in response to infection. The increase was independent of IFN-γ signaling, suggesting that IFN-γ does not affect mucin-release in the nematode infection model. This is quite different from our findings in the infected cecum tissue of *Salmonella* infected mice. Here, infection does not affect mucin mRNA levels, but enhances mucin excretion from goblet cells. The latter is a hallmark of the mucosal *S*. Typhimurium infection [52]. Similar observations have been made in *Listeria monocytogenes*-infected rabbit ileal loops [63]. Nevertheless, even closely related pathogens like “atypical” and “typical” enteropathogenic *E. coli*, can differ significantly in their effects on mucin expression and secretion [64]. Thus, mucin expression and release might be regulated in a pathogen-specific fashion. Such pathogen-specific responses may be of great importance for understanding the host range of different mucosal pathogens and the susceptibility of particular hosts to pathogen-inflicted disease.

We found that IFN-γR-signaling coordinates multiple aspects of mucosal defense, including pathogen restriction in the gut tissue and goblet cell function. Clearly, other cytokines are also involved. Moreover, the epithelial cells of the infected mucosa can produce and secrete anti-microbial peptides and express high levels of the polymeric Ig receptor, a transporter mediating secretory IgA secretion into the gut lumen [65], [66]. Interestingly, this is also controlled by IFN-γ-signaling [9], [67]. Most likely, mucus secretion, antimicrobial peptides and sIgA cooperate to protect the intestinal mucosa. It will be an important topic for future research to unravel how these defenses are coordinated and how this confers efficient protection against gut pathogens.

## Supporting Information

Figure S1 **Pilot experiments showing high numbers of goblet cells with mucus filled vacuoles in the cecal mucosa of infected IFN-γR^-/-^ mice**. IFN-γR-/- mice (C57BL/6 background) and C57BL/6 control mice were pre-treated with streptomycin and infected for 1 day (A; 1dpi) and 2 days (B; 2dpi) with *S*. Typhimurium (SL1344; Material and Methods). The cecal tissue of IFN-γR^-/-^ mice on C57BL/6 background (right panel) seemed to harbor more mucus-filled goblet cells at 1dpi (A) and 2dpi (A) compared to C57BL/6 control mice (left panel). IFN-γR^-/-^ mice (129Sv/Ev background) and 129Sv/Ev control mice were pre-treated with streptomycin and infected for 4 days (C; 4dpi) with *S*. Typhimurium (SL1344; Material and Methods). Again, the cecal mucosa of the IFN-γR^-/-^ animals (right panel) harbored more mucus-filled goblet cells in the cecal tissue compared to 129Sv/Ev control mice (left panel). GHE: mucus-filled goblet cell vacuoles detected in HE-stained tissue sections. Bar  = 100 µm.
(TIFF)
