# Supplementary figures and images for: Stromal IFN-γR-Signaling Modulates Goblet Cell Function During Salmonella Typhimurium Infection
Source: PLoS One. 2011 Jul 28;6(7):e22459. doi: 10.1371/journal.pone.0022459 (PMC3145644; doi:10.1371/journal.pone.0022459)

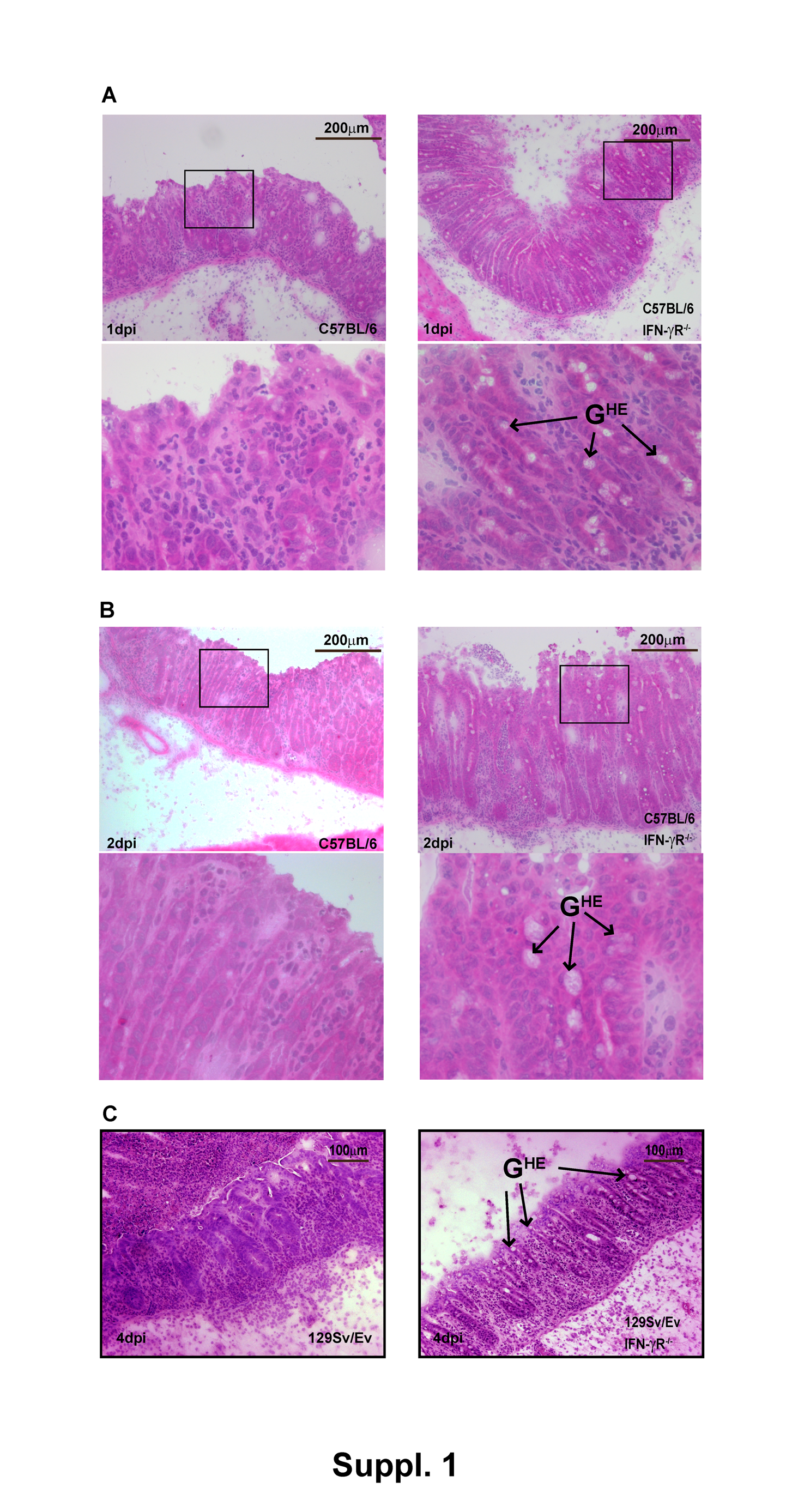

Supplement: Figure S1 — Pilot experiments showing high numbers of goblet cells with mucus filled vacuoles in the cecal mucosa of infected IFN-γR-/- mice. IFN-γR-/- mice (C57BL/6 background) and C57BL/6 control mice were pre-treated with streptomycin and infected for 1 day (A; 1dpi) and 2 days (B; 2dpi) with S. Typhimurium (SL1344; Material and Methods). The cecal tissue of IFN-γR-/- mice on C57BL/6 background (right panel) seemed to harbor more mucus-filled goblet cells at 1dpi (A) and 2dpi (A) compared to C57BL/6 control mice (left panel). IFN-γR-/- mice (129Sv/Ev background) and 129Sv/Ev control mice were pre-treated with streptomycin and infected for 4 days (C; 4dpi) with S. Typhimurium (SL1344; Material and Methods). Again, the cecal mucosa of the IFN-γR-/- animals (right panel) harbored more mucus-filled goblet cells in the cecal tissue compared to 129Sv/Ev control mice (left panel). GHE: mucus-filled goblet cell vacuoles detected in HE-stained tissue sections. Bar = 100 µm. (TIFF) [file pone.0022459.s001.tiff]
